# Effects of Peptide C_12_-OOWW-NH_2_ on Transcriptome and Cell Wall of the Postharvest Fungal Pathogen *Penicillium digitatum*

**DOI:** 10.3389/fmicb.2020.574882

**Published:** 2020-09-17

**Authors:** Xindan Li, Guirong Feng, Wenjun Wang, Lanhua Yi, Lili Deng, Kaifang Zeng

**Affiliations:** ^1^College of Food Science, Southwest University, Chongqing, China; ^2^Research Center of Food Storage & Logistics, Southwest University, Chongqing, China

**Keywords:** *Penicillium digitatum*, peptide, C_12_O_3_TR, RNA-Seq, cell wall

## Abstract

In this study, the transcriptional profiling of *Penicillium digitatum* after C_12_O_3_TR treatment was analyzed by RNA-Seq technology. A total of 2562 and 667 genes in *P. digitatum* were differentially expressed after 2 and 12 h treatment, respectively. These genes were respectively mapped to 91 and 79 KEGG pathways. The expression patterns of differentially expressed genes (DEGs) at 2 and 12 h were similar, mainly were the metabolic processes in cell wall, cell membrane, genetic information and energy. Particularly, the main metabolic process which was affected by C_12_O_3_TR stress for 2 and 12 h was cell integrity, including cell wall and cell membrane. The changes of chitin in cell wall was observed by Calcofluor White (CFW) staining assay. The weaker blue fluorescence in the cell wall septa, the decrease of β-1, 3-glucan synthase activity and the increase of chitinase and AKP activity showed that C_12_O_3_TR could damage the cell wall integrity. In conclusion, these results suggested that C_12_O_3_TR could inhibit the growth of *P. digitatum* through various mechanisms at transcriptional level, and could influence the cell wall permeability and integrity.

## Introduction

During postharvest storage and marketing process, citrus fruit usually suffers significant economic losses mainly due to the green mold disease which was caused by *Penicillium digitatum* (Droby et al., 2008; Lu et al., 2018). Conventional chemical fungicides are highly effective against this pathogen, and therefore are commonly used to control the green mold disease on citrus fruit. However, intense application of chemical fungicides has aroused the concerns to the environment, human health, and development of fungicide resistance strains. Therefore, there is an urgent need to replace or reduce the use of chemical fungicides by effective and eco-friendly methods (Palou et al., 2016; Romanazzi et al., 2015).

Antimicrobial peptides (AMPs) are one of the strong candidates. AMPs are gene-encoded, ribosomally synthesized polypeptides, which are cationic or anionic (Maget-Dana and Peypoux, 1994; Mulder et al., 2013). AMPs are widely present in plants, animals, insects and microorganisms with a broad spectrum of activity against viruses, bacteria, fungi, and parasites (Jenssen et al., 2006). They are also important components against invading pathogens in the biological innate immunity system (López-García et al., 2015; Wang et al., 2018a). Because of their efficient control effects against pathogens, less developmental resistance and low toxicity to host cells, they have been proposed as novel antibiotics in many fields, such as agriculture, animal husbandry and the food industry (Jenssen et al., 2006; Keymanesh et al., 2009; Ciociola et al., 2016). Currently, an increasing number of researchers are trying to use AMPs to control postharvest diseases of fruit and vegetable (Johnson et al., 2015; Puig et al., 2016). There are more and more rationally designed and chemically synthesized peptides which have been proven to be effective against postharvest pathogens, such as BP15, PAF26 and MsrA1 (Osusky et al., 2000; Muñoz et al., 2007; Puig et al., 2016).

Peptide C_12_-OOWW-NH_2_ (C_12_O_3_TR) which was synthesized by combining peptide O_3_TR (H-OOWW-NH_2_) with saturated fatty acids, could effectively inhibit some clinically important bacteria (such as *Staphylococcus epidermidis*, *Staphylococcus aureus*, *Pseudomonas aeruginosa* and *Escherichia coli*) and postharvest pathogens (such as *Penicillium expansum*, *Aspergillus niger*, and *Fusarium culmorum*) (Laverty et al., 2010; Thery et al., 2018). Our previous study showed that C_12_O_3_TR could significantly suppress the growth of *P. digitatum* with the minimum inhibitory concentration (MIC) of 6.25 μmol/L. But the mechanism of C_12_O_3_TR against *P. digitatum* was still unclear. The classic antifungal mechanism mainly focuses on the interaction between AMPs and cell membrane (Abedinzadeh et al., 2015; Omardien et al., 2016). While some researches have proposed other mechanisms which pointed out that some AMPs could interact with intracellular specific targets, such as DNA, RNA and protein or could interfere the synthesis of cell wall to inhibit the growth of pathogens or could induce reactive oxygen species (ROS) production in cell (Petruzzelli et al., 2003; Scocchi et al., 2016; Shah et al., 2016). Recent research has found that C_12_O_3_TR could inhibit the growth of fungi by changing the membrane permeabilization (Li et al., 2019), but whether there are any other antifungal mechanisms needs to be further clarified.

RNA-Seq is an innovative technology for the quantification and identification of gene expression. Due to its sensitivity, high resolution and comprehensive features, it has become increasingly popular in various studies which aim was to reveal the change of the organism gene expression in different environment (Nookaew et al., 2012; OuYang et al., 2016). RNA-seq has also been used to explore the molecular mechanism of fungal drug-resistance or fungi-host interaction (Liu et al., 2015; Barad et al., 2016; Wang et al., 2016). There are several studies that utilized RNA-Seq technology to investigate the fungal response mechanism to peptide such as MAF-1A (Wang et al., 2017).

The objective of this work was to reveal the molecular antifungal mechanism of C_12_O_3_TR against *P. digitatum* by using high-throughput RNA-Seq technique, and to confirm the effect of C_12_O_3_TR on cell wall at superficial level.

## Materials and Methods

### Fungal Species

*Penicillium digitatum* was used in this work and cultured on potato dextrose agar (PDA) plates at 25°C (Wang et al., 2018b; Liu S. et al., 2019; Liu Y. et al., 2019). Fungal conidia suspension was obtained by flooding the 7-days-old culture spores with sterile distilled water, followed by filtering through four layers of sterile gauze, and then adjusting to the suitable concentration.

### Peptide Synthesis

The peptide (C_12_H_23_O)-OOWW-NH_2_ (C_12_O_3_TR) was purchased from GenScript Corporation (Nanjing, China) with > 90% purity. The purity was selected based on other relevant studies and cost consideration (Laverty et al., 2015; Thery et al., 2018). Peptide C_12_O_3_TR was synthesized by the solid-phase methods using 9-fluorenylmethoxy carbonyl (Fmoc)-type chemistry. C_12_O_3_TR was amidated at the C terminus. Stock solutions of peptide was reconstituted at 1 mmol/L by using sterile distilled water (C_12_O_3_TR could completely dissolved by using sterile distilled) and then stored at −40°C. C_12_O_3_TR was not sensitive to oxidation.

### Preparation of C_12_O_3_TR Treatments

*P. digitatum* conidia (1 × 10^5^CFU/mL, 100 μL) were inoculated in 20 mL potato dextrose broth (PDB), and incubated at 25°C in thermostatic shaker at 160 rpm for 2 d. The *P. digitatum* mycelia were obtained by centrifuging at 4000 × g for 15 min. After washing with PBS (pH 7.0) 3 times, the mycelia were resuspended in 20 mL PBS (pH 7.0). Subsequently, the peptide C_12_O_3_TR was added into the suspensions to the final concentration of 6.25 μmol/L (MIC), and then incubated at 25°C for 0, 2, 4, 6, and 12 h. PBS (pH 7.0) was used as the control. Each treatment was repeated three times. Finally, the mycelia samples which removed PBS were immediately frozen in liquid nitrogen and stored at -80°C until use.

### RNA Extraction and Illumina Sequencing

The *P. digitatum* mycelia samples after 2 and 12 h of C_12_O_3_TR and PBS treatment were used for this experiment. The four treatments were named C2, C_12_O_3_TR2, C12, C_12_O_3_TR12, respectively. Total RNA preparation, RNA quality detection, cDNA libraries construction and RNA-seq were carried out by using a service from Novogene Bioinformatics Technology Co., Ltd. (Beijing, China). Total RNA was extracted by using TRIzol reagent (Invitrogen, United States) according to the manufacturer’s instructions. RNA contamination and degradation were monitored on 1% agarose gels. The NanoPhotometer^®^ spectrophotometer (Implen, CA, United States) was used to check RNA purity. RNA integrity was assessed by using the RNA Nano 6000 Assay Kit of the Bioanalyzer 2100 system (Agilent Technologies, CA, United States) (Wang et al., 2017).

One μg RNA per sample was used as input material for the RNA sample preparations. NEBNext^®^ UltraTM RNA Library Prep Kit for Illumina^®^ (NEB, United States) following manufacturer recommendations was used to generate sequencing libraries and index codes were added to attribute sequences to each sample. Then the cDNA libraries were sequenced by using an Illumina HiSeq2000^TM^ platform (Lin et al., 2013; OuYang et al., 2016; Lai et al., 2017). The resulting RNA-seq reads were mapped onto the reference genome of *Penicillium digitatum* Pd1 (GCA_000315645)^1^ (Marcet-Houben et al., 2012). Gene model annotation files and reference genome were directly downloaded from genome website.^2^ Index of the reference genome was built by using Hisat2 v2.0.5. Paired-end clean reads were aligned to the reference genome by using Hisat2 v2.0.5.

In order to identify differentially expressed genes (DEGs) between C2 and C_12_O_3_TR2, or C12 and C_12_O_3_TR12, transcript abundance was estimated by using the method of expected number of Fragments Per Kilobase of transcript sequence per Millions base pairs sequenced (FRKM) (Trapnell et al., 2010). Differential expression analysis of two conditions/groups (three biological replicates per condition) was performed by using the DESeq R package (??). The *P*-values were adjusted by using the Benjamini and Hochberg method. *P*-value of 0.05 and absolute fold change of 2 were set as the threshold for significantly differential expression (Wang et al., 2010).

Gene Ontology (GO) enrichment analysis of DEGs were implemented by using the cluster Profiler R package. GO terms with corrected *P*-value (padj) < 0.05 were considered significantly enriched (Young et al., 2010). In order to identify the biological pathways which were active in *P. digitatum*, all DEGs were mapped to the reference canonical pathways contained in the Kyoto Encyclopedia of Genes and Genomes (KEGG) pathway database.^3^ The statistical enrichment of DEGs in KEGG pathways were tested by using cluster Profiler R package (Mao et al., 2005). The two-tailed Fisher exact test based on the false discovery rate (FDR) cutoff of 0.05 was used as one of the justification conditions.

### Quantitative Real-Time PCR (qRT-PCR) Analysis

Ten DEGs were selected in this study to validate the results of RNA-Seq. The RNA, which used for quantitative reverse transcription PCR (qRT-PCR) analysis, was extracted by using the same method of 2.3 and qualified by Nanodrop 2000 Spectrophotometer (Thermo-Fisher scientific Inc., Wilmington, DE, United States). cDNAs were constructed from 1 μg total RNA by reverse transcription using the PrimeScript^®^ RT Reagent Kit with gDNA Eraser (TAKARA, Tokyo, Japan). qRT-PCR analysis was performed as described by Zhou Y. et al. (2018), with some modifications. Briefly, 20 μL reaction system of SYBR Green PCR Master Mix (Applied Biosystems, United States) and the Step One Plus Real-time PCR System (Applied Biosystems, United States) were used for the qRT-PCR analysis. Reaction procedures were started at 95°C for 30 s, followed by 40 cycles of 95°C for 10 s and then hold at 60°C for 30 s. The primers for the qRT-PCR were synthesized by Sangon Biotech (Shanghai, China) and presented in Supplementary Table S1. The changes in SYBR Green fluorescence in every cycle were monitored, and the threshold cycle (Ct) over the background were calculated for each reaction. The relative expression level of the genes were calculated using the 2 ^(–ΔΔ Ct)^ method (Livak and Schmittgen, 2001). The aim of experiment was mainly qualitatively verified the reliability of the RNA-Seq data by qRT-PCR. The actin gene (PDIP_27720) was utilized as the internal reference to normalize the expression data (OuYang et al., 2016). Each PCR reaction was repeated three times, and there were three parallel sets for each reaction.

### Assays for Alkaline Phosphatase (AKP) Activity

The AKP activity of *P. digitatum* mycelia was determined by AKP kit (Solarbio Science and Technology Co., Ltd., Beijing, China). The *P. digitatum* mycelia samples after 0, 2, 4, 6, and 12 h of C_12_O_3_TR treatment were used for this experiment. PBS (pH 7.0) was used as the control. Each experiment was repeated three times. One unit of AKP activity was defined as the time (min) to produce 1 μmol phenol per 1 g *P. digitatum* mycelia sample at 37°C.

### Assays for β-1, 3-Glucan Synthase Activity

β-1, 3-glucan synthase activity was analyzed as described by Moreno-Velásquez et al. (2017), with some modifications. Four hundred mg *P. digitatum* mycelia samples were homogenized with 0.1 mol/L citrate - 0.2 mol/L disodium phosphate buffer (pH 4.8, 1.5 mL) in liquid nitrogen. The mixture was then centrifuged at 12000 × *g* for 10 min at 4°C, and the supernatant was collected as the enzyme extract liquid. After that, 1 mL enzyme extract liquid and 1 mL 0.1 mol/L citrate - 0.2 mol/L disodium phosphate buffer (pH 4.8) were mixed with fucoidan solution (1 mg of fucoidan dissolved in 1 mL distilled water), and then the solution was incubated at 30°C water bath for 1 h. Then, distilled water (1.5 mL), anthrone ethyl acetate (0.5 mL) and H_2_SO_4_ (3 mL) were respectively orderly added in 0.5 mL solution which was after 30°C water bath. The whole mixture was heated in boiling water bath for 1 min, and then cooled down to room temperature to measure the absorbance at 630 nm. One unit of β-1, 3-glucan synthase activity was defined as the time (hour) to produce 1 mg glucose per 1 g *P. digitatum* mycelia sample at 30°C. The glucose content standard curve was calculated based on glucose content (x axis) against absorbance value (y axis) (y = 1.662x + 0.1545, R^2^ = 0.9904).

### Assays for Chitinase Activity

Chitinase activity was analyzed as described by Pan et al. (2020), with some modifications. The samples after 0, 2, 4, 6, and 12 h of C_12_O_3_TR treatment were used for this experiment. Two hundred mg *P. digitatum* mycelia samples were homogenized in liquid nitrogen with acetic acid buffer (1 mL), and then centrifuged at 12000 × *g* for 10 min at 4°C. The supernatant was collected as enzyme extract liquid. The enzyme extract liquid (0.4 mL) and acetic acid buffer (0.4 mL) were mixed with colloidal chitin solution, and then the mixture was incubated at 37°C water bath for 2 h. The reaction system was stopped by centrifuging at 4000 × *g* for 10 min. Afterward, 0.4 mL of the supernatant was mixed with saturated borax solution (0.2 mL), and then the mixture was heated in boiling water bath for 7 min. After cooling to room temperature, glacial acetic acid (2 mL) and 1% p-dimethylaminobenzaldehyde (DMAB) (1 mL) were added in the solution which was cooled to room temperature. The absorbance was measured at 585 nm, and the chitinase activity was calculated based on the standard curve. One unit of chitinase activity was defined as the time (hour) to produce 1 μg of N-acetylglucosamine (GlcNAc) per 1 g of *P. digitatum* mycelia at 37°C. The standard curve of GlcNAc content was established according to GlcNAc content (x axis) against the absorbance value (y axis) (y = 0.0115x + 0.0359, R^2^ = 0.9974).

### The Effect of C_12_O_3_TR on the Cell Wall Integrity of *P. digitatum*

The effect of C_12_O_3_TR on *P. digitatum* cell wall integrity was determined as described by OuYang et al. (2019), with some modifications. *P. digitatum* conidia (1 × 10^4^ CFU/mL, 90 μL) mixed with 5% PDB were cultured at 25°C for 48 h. C_12_O_3_TR with the final concentrations of 6.25 μmol/L (MIC) was added and then incubated for 0, 2, and 12 h. PBS (pH 7.0) was used as control. Each sample was then stained with 50 mg/L Calcofluor White (CFW) for 5 min in dark, and the fluorescence was examined and photographed by the Eclipse TS100 epifluorescence microscope (Nikon Corporation, Japan) with DAPI filter sets. The reproducibility of experiment results was confirmed by three replicates.

### Statistical Analysis

All experiments included three parallel sets and were repeated three times. The data were processed with Microsoft Excel 2013, and analyzed by statistical software SPSS 21.0 (SPSS Inc., Chicago, IL, United States). The variance of data was analyzed via one-way analysis of variance (ANOVA) with Duncan’s multiple range tests at *p* < 0.05.

## Results

### RNA Sequencing and Gene Prediction

The profile of transcriptome sequence average data was shown in Table 1. The objective data for sequencing each sample was shown in Supplementary Table S2. Based on RNA-seq, an average of 63.93 ± 4.00 million, 66.06 ± 6.00 million, 58.83 ± 2.72 million and 57.94 ± 2.26 million raw reads were generated from C2, C_12_O_3_TR2, C12, and C_12_O_3_TR12 samples, respectively. After filtering the adaptor sequences, the average clean reads were 62.50 ± 4.62 million, 64.98 ± 5.89 million, 57.76 ± 2.65 million and 56.47 ± 1.86 million for the four treatment groups, respectively. Among them, 94.87 ± 0.08%, 95.06 ± 0.22%, 95.23 ± 0.10% and 95.21 ± 0.16% of the total reads were mapped to the genome of *P. digitatum* in C2, C_12_O_3_TR2, C12, and C_12_O_3_TR12 samples, respectively. In Supplementary Table S2, the percentages of the 12 sequencing samples total reads mapped to the genome of *P. digitatum* were all more than 90%. And 94.39 ± 0.10%, 94.60 ± 0.22%, 94.66 ± 0.08% and 94.61 ± 0.14% of the reads were uniquely mapped. In addition, the mapped reads of 12 samples represented the filtered data were all less than 1%, in Supplementary Table S2. In conclusion, none of the sequencing samples were contaminated, and all the experimental samples met the requirements of subsequent experiments. *3.2. Transcriptional Stress Response of P. digitatum to peptide C_12_O_3_TR.*

**TABLE 1:** Profile of the transcriptome sequence data.

Parameter	C2	C_12_O_3_TR2	C12	C_12_O_3_TR12
Raw reads (million)	63.93 ± 4.00	66.06 ± 6.00	58.83 ± 2.72	57.94 ± 2.26
Clean reads (million)	62.50 ± 4.62	64.98 ± 5.89	57.76 ± 2.65	56.47 ± 1.86
Clean bases (G)	9.38 ± 0.69	9.75 ± 0.89	8.67 ± 0.40	8.47 ± 0.28
Error rate (%)	0.02 ± 0.00	0.03 ± 0.00	0.02 ± 0.00	0.02 ± 0.00
Q20 (%)	98.14 ± 0.22	97.97 ± 0.27	98.33 ± 0.02	98.31 ± 0.06
Q30 (%)	94.63 ± 0.45	94.33 ± 0.55	95.05 ± 0.04	94.96 ± 0.14
GC content (%)	53.78 ± 0.00	53.62 ± 0.10	53.64 ± 0.07	53.58 ± 0.06
Total mapped reads	59291298 (94.87 ± 0.08%)	61780893 (95.06 ± 0.22%)	55008480 (95.23 ± 0.10%)	53768470 (95.21 ± 0.16%)
Uniquely mapped reads	58985616 (94.39 ± 0.10%)	61483597 (94.60 ± 0.22%)	54675270 (94.66 ± 0.08%)	53428164 (94.61 ± 0.14%)
Multiple mapped reads	305682 (0.49 ± 0.02%)	297296 (0.46 ± 0.01%)	333210.7 (0.57 ± 0.02%)	340306.3 (0.60 ± 0.05%)

*The samples C2 was control group, and the C_12_O_3_TR2 samples belonged to the group exposed to peptide C_12_O_3_TR at MIC after 2 h. Similarly, C12 was control group, and C_12_O_3_TR12 was the group which exposed to C_12_O_3_TR at MIC after 12 h. Q20 and Q30 are the percentage of quality values greater than or equal to 20 or 30.

**FIGURE 1:**
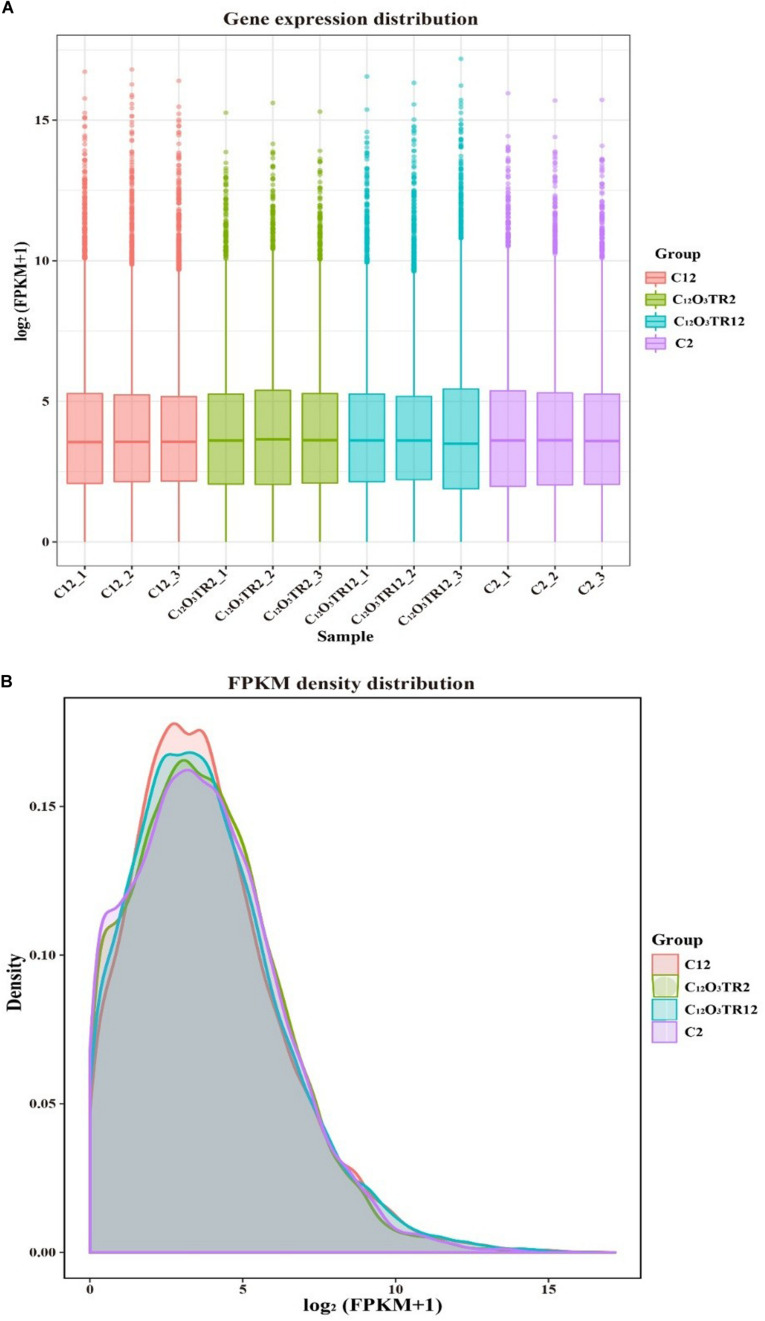
The comparison diagram of gene expression level. **(A)** Gene expression distribution. The x-axis shows the name of samples, and C2-1, C2-2 and C2-3 represent three biological duplicate. The y-axis shows the value of log_2_^(FPKM+1)^. **(B)** FPKM density distribution. The x-axis shows the value of log_2_^(FPKM+1)^. The y-axis shows the density of samples.

**FIGURE 2:**
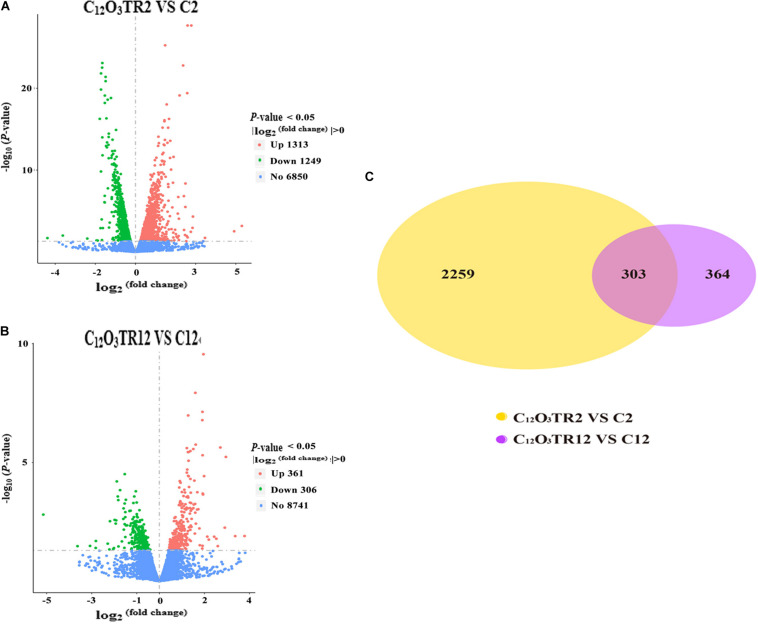
The volcano plots of the DEGs and gene expression Venn diagram. Volcano plots of DEGs between *P. digitatum* under C_12_O_3_TR (MIC) treatment after 2 h **(A)** and 12 h **(B)**. The x-axis shows the fold change in gene expression, and the y-axis shows the statistical significance of the differences. Splashes represent different genes. Blue dots indicate genes without significant differential expression. Red dots mean significantly up-regulated genes. Green splashes mean significantly down-regulated genes. **(C)** The number in each circle represents the total number of DEGs that are expressed in 2 h (between C12O3TR2 and C2) and 12 h (between C12O3TR12 and C12) datasets, respectively. The overlapping part of circles indicates that the gene is co-expressed in both 2 and 12 h.

The RNA sequencing results in Figure 1 revealed the differences in distribution and density distribution of gene expression in C2, C_12_O_3_TR2, C12 and C_12_O_3_TR12 samples. There were also differences in gene expression distribution among three biological replicates under the same treatment (Figure 1A). The gene expressions of all groups had the same characteristics, which were, most of the genes were low in expression, while a few genes were high in expression (Figure 1B). In conclusion, these phenomena were consistent with the law of gene expression in biology. The volcano plots of the DEGs demonstrated that there were 2562 genes which were differentially expressed in *P. digitatum* after 2 h C_12_O_3_TR treatment, including 1313 up-regulated genes and 1249 down-regulated genes (Figure 2A). In addition, a total of 667 DEGs were detected between C12 and C_12_O_3_TR12, in which 361 genes were up-regulated and 306 were down-regulated (Figure 2B). To further analyze the effect of different time on gene expression of *P. digitatum* mycelia in response to C_12_O_3_TR, DEGs in groups treated with C_12_O_3_TR for 2 and 12 h were compared. The results showed that 303 genes were consistently differentially expressed in the two treatments (Figure 2C).

### Enrichment Analysis of GO

**FIGURE 3:**
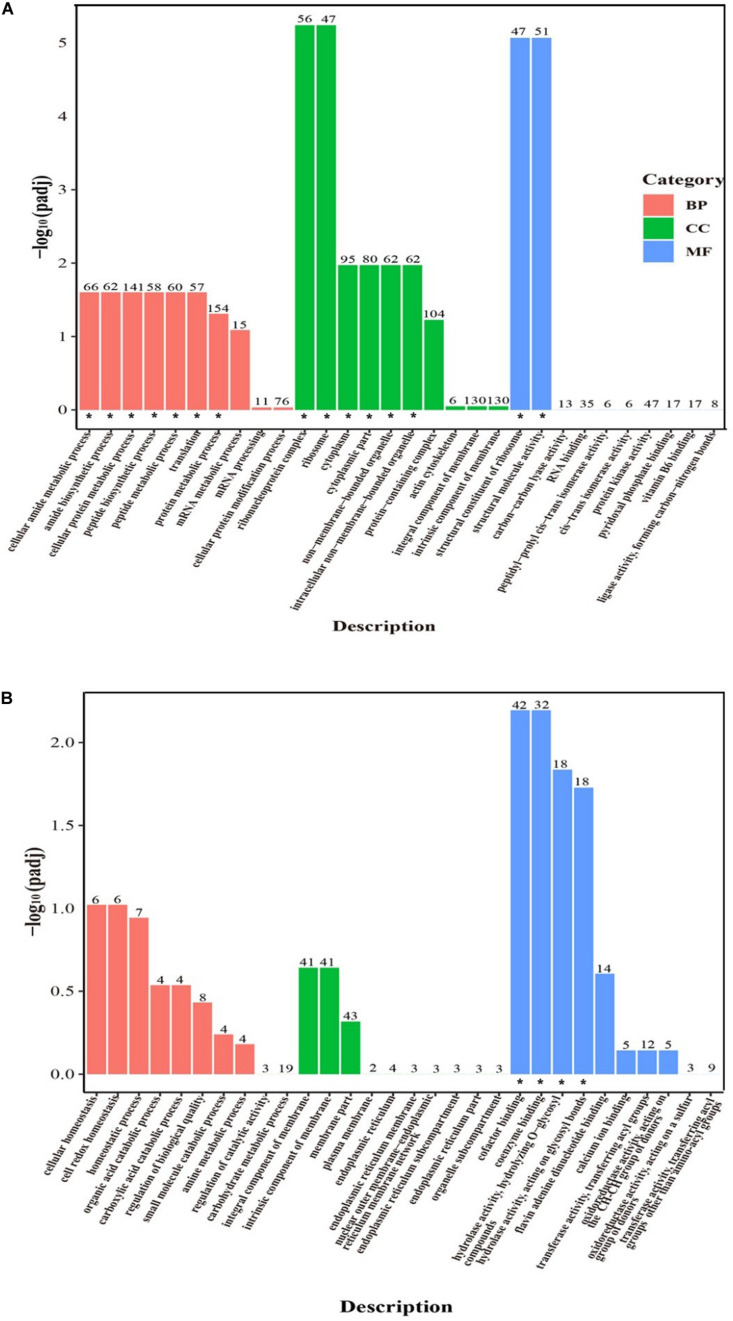
GO functional classification analysis of DEGs. GO functional classification of DEGs in *P. digitatum* under C12O3TR (MIC) stress after 2 h **(A)** and 12 h **(B)** of culturing. X axis means GO term. Y axis represents the value of –log10 (padj). The number on each pillar is the number of DEGs in each GO term. All GO terms are grouped into three ontologies: red is for biological process (BP), green is for cellular component (CC), and blue is for molecular function (MF). The “*” symbol located at the figure indicated that the GO term was significantly enriched (padj < 0.05).

GO analysis was used to classify the DEGs of *P. digitatum* in response to C_12_O_3_TR. A total of 2562 DEGs were mapped to 572 GO terms in sample treated with C_12_O_3_TR for 2 h. Among them, 308, 74, and 190 GO terms were assigned to biological process, cellular component and molecular function, respectively. Figure 3A showed the top 30 enriched functional categories of the 2562 DEGs. The part information of the significant enrichment terms were shown in the Supplementary Table S3. As could be seen, the significant enrichment terms in biological process included cellular amide metabolic process, amide biosynthetic process, cellular protein metabolic process, peptide biosynthetic process, peptide metabolic process, translation and protein metabolic process. Significant enrichment terms in cellular component were ribonucleoprotein complex, ribosome, cytoplasm, cytoplasmic part, non-membrane-bounded organelle and intracellular non-membrane-bounded organelle. And the significant enrichment terms in molecular function were mostly structural constituent of ribosome and structural molecule activity.

After 12 h C_12_O_3_TR treatment, 667 DEGs were mapped to 572 GO terms (Figure 3B), in which 201, 46, and 165 GO terms were assigned to biological process, cellular component and molecular function, respectively. The part information of the significant enrichment terms were presented in the Supplementary Table S4. In biological process, main functional terms were cellular homeostasis, cell redox homeostasis, homeostatic process, organic acid catabolic process, carboxylic acid catabolic process and regulation of biological quality among others; cellular component mainly enriched in terms of integral component of membrane, intrinsic component of membrane, membrane part, plasma membrane, endoplasmic reticulum and endoplasmic reticulum membrane among others. And significant enrichment terms in molecular function were cofactor binding, coenzyme binding, hydrolase activity, hydrolyzing O-glycosyl compounds and hydrolase activity, acting on glycosyl bonds.

### Enrichment Analysis of KEGG Pathways

For KEGG analysis, the DEGs were mapped to 91 and 79 KEGG pathway in samples treated with C_12_O_3_TR for 2 and 12 h, respectively. The 50 most enriched pathways were shown in Table 2. The two groups had the same expression patterns, mainly in cell wall, cell membrane, genetic information and energy metabolic pathways. In detail, the pathways which were associated with cell wall were fructose and mannose metabolism, amino sugar and nucleotide sugar metabolism; cell membrane metabolic pathways were mostly sphingolipid metabolism, glycerophospholipid metabolism, glycerolipid metabolism, inositol phosphate metabolism, glycosylphosphatidylinositol (GPI)-anchor biosynthesis, ether lipid metabolism, fatty acid biosynthesis, biosynthesis of unsaturated fatty acids, steroid biosynthesis, fatty acid metabolism and fatty acid degradation; genetic information pathways were ribosome, spliceosome, SNARE interactions in vesicular transport (The transport membrane bubble must fuse with the target membrane during transport to achieve the purpose of transport. A model to explain the mechanism at the molecular level is known as the SNARE hypothesis.), RNA degradation, protein processing in endoplasmic reticulum, proteasome and protein export; and the pathways which were related to energy metabolism were citrate cycle (TCA cycle), sulfur metabolism and nitrogen metabolism. In addition, there were difference between the expression patterns of 2 and 12 h. The genes of ribosome, spliceosome and RNA degradation which were related to genetic information processing were only differentially expressed in 2 h. Interestingly, the genes of protein processing in endoplasmic reticulum, proteasome and protein export which were also in connection with genetic information processing were only differentially expressed in 12 h.

**TABLE 2:** Mostly enriched KEGG pathway of DEGs in *P. digitatum*.

2 h				12 h			
Pathway	Input number	Background number	Pathway ID	Pathway	Input number	Background number	Pathway ID
Ribosome	44	82	pcs03010	Glycine, serine and threonine metabolism	11	38	pcs00260
Citrate cycle (TCA cycle)	14	26	pcs00020	beta-Alanine metabolism	8	21	pcs00410
Pyruvate metabolism	16	32	pcs00620	Valine, leucine and isoleucine degradation	9	32	pcs00280
Carbon metabolism	41	102	pcs01200	Glyoxylate and dicarboxylate metabolism	8	36	pcs00630
beta-Alanine metabolism	11	21	pcs00410	Biosynthesis of secondary metabolites	31	302	pcs01110
Glyoxylate and dicarboxylate metabolism	16	35	pcs00630	Amino sugar and nucleotide sugar metabolism	9	48	pcs00520
Nitrogen metabolism	8	15	pcs00910	Tyrosine metabolism	7	31	pcs00350
Alanine, aspartate and glutamate metabolism	13	29	pcs00250	Cysteine and methionine metabolism	8	43	pcs00270
Sphingolipid metabolism	8	16	pcs00600	Glutathione metabolism	6	26	pcs00480
Glycine, serine and threonine metabolism	16	38	pcs00260	Biosynthesis of unsaturated fatty acids	4	12	pcs01040
Spliceosome	32	85	pcs03040	Alanine, aspartate and glutamate metabolism	6	29	pcs00250
Fructose and mannose metabolism	12	28	pcs00051	Fatty acid metabolism	6	29	pcs01212
Pantothenate and CoA biosynthesis	9	20	pcs00770	Propanoate metabolism	5	22	pcs00640
Ether lipid metabolism	6	12	pcs00565	Arginine and proline metabolism	6	32	pcs00330
SNARE interactions in vesicular transport	6	12	pcs04130	Protein processing in endoplasmic reticulum	9	70	pcs04141
Glutathione metabolism	11	26	pcs00480	Phenylalanine metabolism	4	22	pcs00360
Valine, leucine and isoleucine degradation	13	32	pcs00280	Carbon metabolism	11	103	pcs01200
2-Oxocarboxylic acid metabolism	14	35	pcs01210	Biosynthesis of antibiotics	20	231	pcs01130
N-Glycan biosynthesis	11	27	pcs00510	Fructose and mannose metabolism	4	28	pcs00051
Methane metabolism	8	19	pcs00680	Peroxisome	5	42	pcs04146
Phenylalanine metabolism	9	22	pcs00360	Tryptophan metabolism	4	31	pcs00380
Amino sugar and nucleotide sugar metabolism	17	48	pcs00520	Fatty acid degradation	3	20	pcs00071
Pentose phosphate pathway	9	24	pcs00030	Steroid biosynthesis	3	21	pcs00100
Fatty acid biosynthesis	5	12	pcs00061	Fatty acid biosynthesis	2	12	pcs00061
Thiamine metabolism	5	12	pcs00730	Ether lipid metabolism	2	12	pcs00565
Pentose and glucuronate interconversions	8	21	pcs00040	Histidine metabolism	2	13	pcs00340
Biosynthesis of secondary metabolites	95	301	pcs01110	Various types of N-glycan biosynthesis	3	25	pcs00513
Cysteine and methionine metabolism	15	43	pcs00270	Inositol phosphate metabolism	3	26	pcs00562
Tryptophan metabolism	11	31	pcs00380	Glycerophospholipid metabolism	4	39	pcs00564
Propanoate metabolism	8	22	pcs00640	N-Glycan biosynthesis	3	27	pcs00510
Glycosylphosphatidylinositol (GPI)-anchor biosynthesis	7	19	pcs00563	Nitrogen metabolism	2	15	pcs00910
Valine, leucine and isoleucine biosynthesis	6	16	pcs00290	Valine, leucine and isoleucine biosynthesis	2	16	pcs00290
Porphyrin and chlorophyll metabolism	6	16	pcs00860	Butanoate metabolism	2	17	pcs00650
Peroxisome	14	42	pcs04146	Cyanoamino acid metabolism	2	18	pcs00460
RNA degradation	16	49	pcs03018	Methane metabolism	2	19	pcs00680
Phagosome	11	33	pcs04145	Lysine degradation	2	20	pcs00310
Arginine biosynthesis	6	17	pcs00220	Glycerolipid metabolism	2	23	pcs00561
Folate biosynthesis	6	17	pcs00790	Glycolysis/Gluconeogenesis	3	39	pcs00010
One carbon pool by folate	5	14	pcs00670	Ubiquinone and other terpenoid-quinone biosynthesis	1	11	pcs00130
Biosynthesis of amino acids	34	109	pcs01230	Thiamine metabolism	1	12	pcs00730
Cyanoamino acid metabolism	6	18	pcs00460	SNARE interactions in vesicular transport	1	12	pcs04130
Various types of N-glycan biosynthesis	8	25	pcs00513	One carbon pool by folate	1	14	pcs00670
Biosynthesis of unsaturated fatty acids	4	12	pcs01040	Pyruvate metabolism	2	32	pcs00620
Fatty acid metabolism	9	29	pcs01212	Proteasome	2	34	pcs03050
Inositol phosphate metabolism	8	26	pcs00562	Nicotinate and nicotinamide metabolism	1	16	pcs00760
Autophagy - yeast	19	64	pcs04138	Sulfur metabolism	1	16	pcs00920
Fatty acid degradation	6	20	pcs00071	Protein export	1	16	pcs03060
Phenylalanine, tyrosine and tryptophan biosynthesis	6	20	pcs00400	MAPK signaling pathway - yeast	3	54	pcs04011
Tyrosine metabolism	9	31	pcs00350	Arginine biosynthesis	1	17	pcs00220
Riboflavin metabolism	3	10	pcs00740	Folate biosynthesis	1	17	pcs00790

*The pathway enrichment statistics of DEGs in *P. digitatum* under C_12_O_3_TR (MIC) stress after 2 and 12 h of culturing. The input number is the DEGs with pathway annotation. The background number is all genes with pathway annotation.*

### Verification of the Expression Level of Candidate DEGs

**FIGURE 4:**
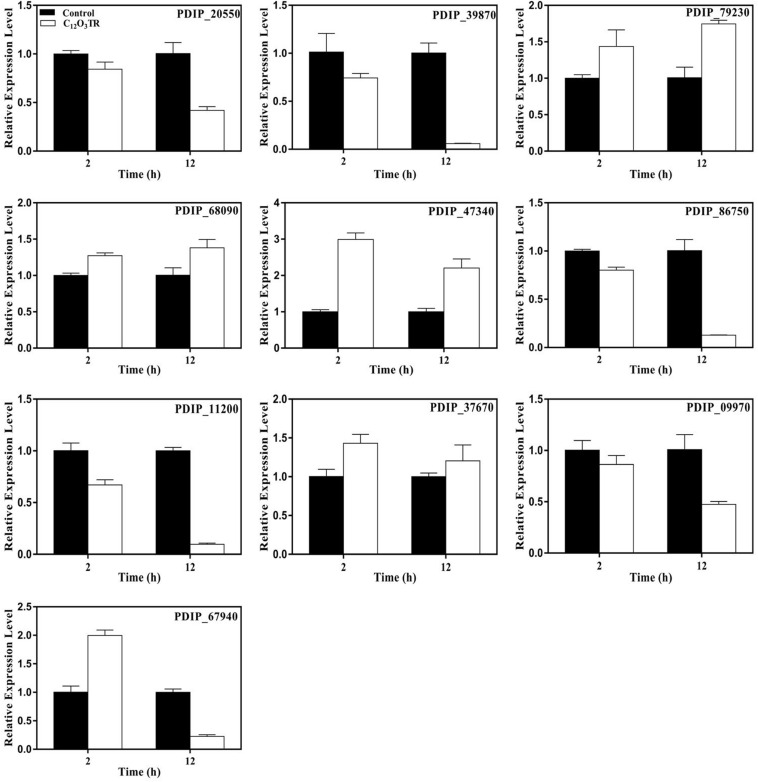
Relative expression levels of candidate DEGs by quantitative real-time PCR. The actin gene is used as the internal control. Samples were prepared in triplicate, and the bars indicate standard error. Each PCR reaction was carried out in triplicate for three repeats. Columns and bars represent the means and standard error (*n* = 3), respectively.

A total of ten genes which were related to cell membrane and cell wall metabolisms and were simultaneous differently expressed in both 2 and 12 h were selected to validate the RNA-Seq results (Supplementary Table S1). The results of qRT-PCR experiments revealed that the genes showed the same expression profile as the RNA-Seq data, and then confirmed the reliability of the RNA-Seq data (Figure 4).

### Effect of C_12_O_3_TR on the Cell Wall Integrity of *P. digitatum*

**FIGURE 5:**
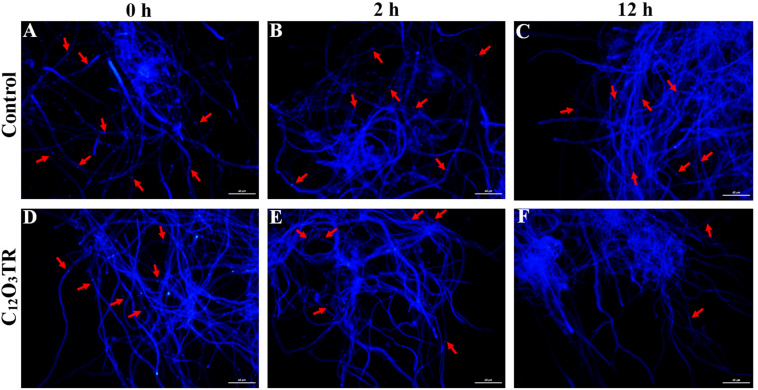
The effects of C_12_O_3_TR on the cell wall integrity of *P. digitatum*. The *P. digitatum* mycelia were treated without C_12_O_3_TR **(A–C)** or with MIC of C_12_O_3_TR after 0 h **(D)**, 2 h **(E)**, and 12 h **(F)**, respectively. Bars = 60 μm.

**FIGURE 6:**
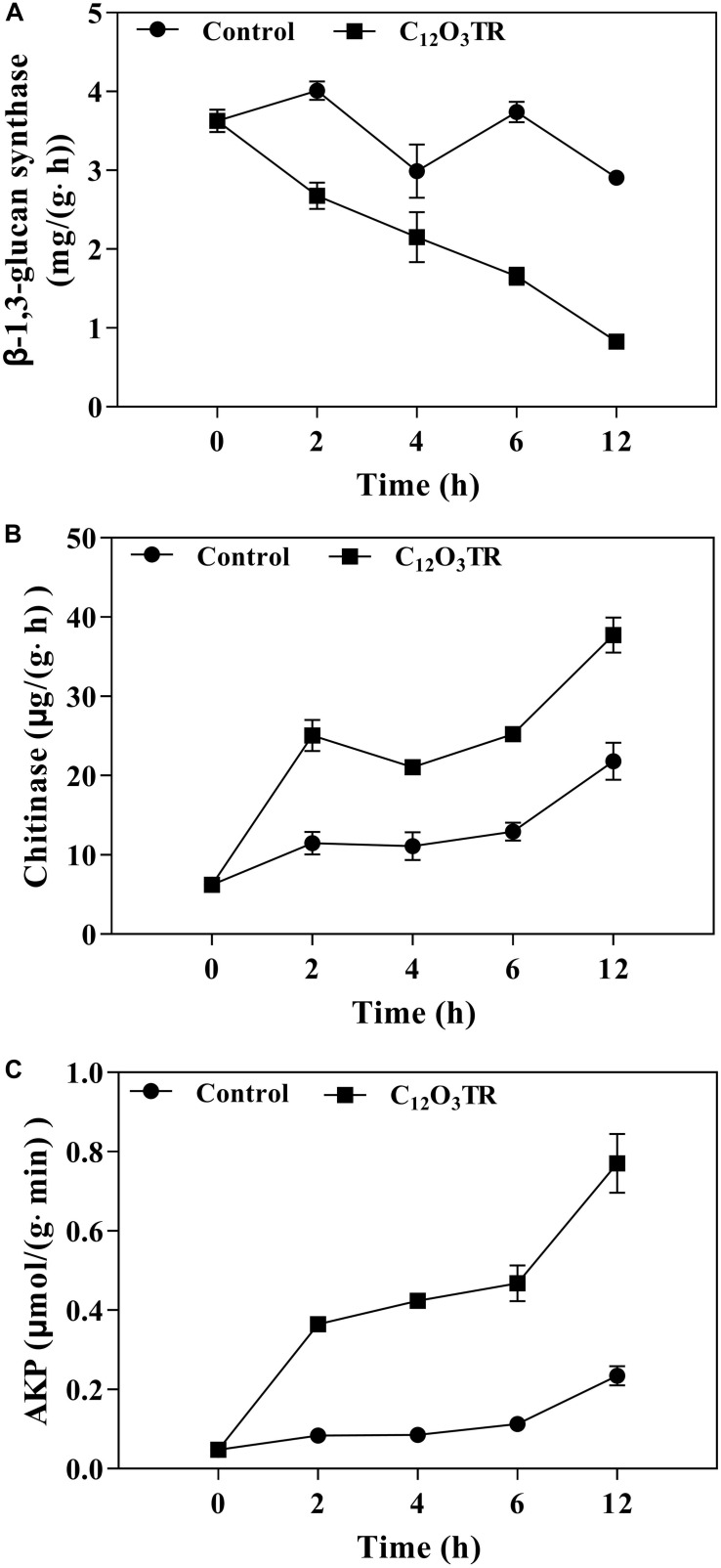
Effect of C_12_O_3_TR on β-1, 3-glucan synthase **(A)**, chitinase **(B)**, and extracellular AKP **(C)** activities in *P. digitatum*. Mycelia were mixed with C_12_O_3_TR at MIC or without peptide in PBS (pH 7.0). Samples were prepared in triplicate, and the bars indicate the standard error of the means.

Figure 6 exhibited the effect of C_12_O_3_TR on the cell wall integrity of *P. digitatum.* When mycelia were stained with CFW for 0, 2, and 12 h without C_12_O_3_TR, almost every septa were visible with bright blue fluorescent lines because of the high chitin content therein (Figures 5A–C). However, the weaker and fewer blue fluorescence septa were observed in C_12_O_3_TR treated groups than that in control group when treated with the same time (Figures 5D–F). And the number of blue fluorescent septa gradually decreased along with the C_12_O_3_TR treatment time. Almost no blue fluorescence septa could be observed in after 12 h of C_12_O_3_TR treatment groups (Figure 5F).

### Effect of C_12_O_3_TR on Cell Wall Related Enzymes Activities in *P. digitatum*

The influence of C_12_O_3_TR on enzyme activities related to cell wall metabolism of *P. digitatum* were shown in Figure 6. The β-1, 3-glucan synthase is the vital enzyme which controlled the synthesis of glucan in cell wall. In this study, the β-1, 3-glucan synthase activity was decreased along with the treatment time (Figure 6A). C_12_O_3_TR significantly reduced the β-1, 3-glucan synthase activity which was 0.83 ± 0.04 mg/(g ⋅ h) when treated with C_12_O_3_TR for 12 h, while the activity was still 2.91 ± 0.01 mg/(g ⋅ h) in control group.

The chitinase is the vital enzyme which effectively catalytic hydrolysis of chitin in cell wall. For chitinase activity, C_12_O_3_TR treated group showed higher chitinase activity than control group (*P* < 0.05), and the chitinase activity also increased with the C_12_O_3_TR treatment time (Figure 6B). At 12 h, the chitinase activity in C_12_O_3_TR treatment was 37.74 ± 1.79 μg/(g h), which was significantly higher than that in control group [21.79 ± 1.93 μg/(g ⋅ h)].

AKP is produced in the cytoplasm and leaked into the periplasmic space. Generally, AKP releases from fungal cells with impaired cell wall permeability. In our study, C_12_O_3_TR also increased the AKP activity in *P. digitatum* (Figure 6C). After 2 h of treatment, the AKP activity in C_12_O_3_TR group was 0.08 ± 0.00 μmol/(g ⋅min), yet it was 0.05 ± 0.01 μmol/(g ⋅min) in control group. This change became more evident when treated with longer time (*P* < 0.05). At 12 h, the extracellular AKP activity in the C_12_O_3_TR treatment was 0.77 ± 0.07 μmol/(g ⋅min), which was significantly higher than that in control group [0.23 ± 0.02 μmol/(g min)].

## Discussion

Our previous study found that peptide C_12_O_3_TR was effective to inhibit the growth of *P. digitatum*. Therefore C_12_O_3_TR was useful to control the green mold on citrus fruit (Li et al., 2019). The present works aimed to further investigate the possibly antifungal mechanism of C_12_O_3_TR against *P. digitatum* by transcriptomic profile determination through RNA-Seq analysis. Our results showed that C_12_O_3_TR significantly influenced a large number of gene expression and metabolic processes. The expression patterns of DEGs at 2 and 12 h were similar, which included the metabolic processes in cell wall, cell membrane, genetic information and energy. However, the expression of pathways, which were related to genetic information processing, were different in 2 and 12 h. The difference shown that the effect of C_12_O_3_TR on *P. digitatum* was different with the treat time owing to differential cellular statuses. This result was similar with the effect of essential oil decanal on the postharvest fungal pathogen *Penicillium expansum* in different time (Zhou T. et al., 2018).

Cell membrane plays an important role in maintaining the cell viability because it is a barrier that separates the cell from its surroundings, and is a channel for exchanging substances and energy between the cell and the surrounding environment (Shao et al., 2013; Tao et al., 2014). Its integrity is highly related to many metabolic processes. In this study, C_12_O_3_TR affected multiple cell membrane related metabolic processes, such as fatty acid biosynthesis, biosynthesis of unsaturated fatty acids, steroid biosynthesis, fatty acid metabolism and fatty acid degradation among others (Table 2). These results indicated that C_12_O_3_TR disrupted the normal metabolic processes of the cell membrane, and finally damaged the cell member integrity. In addition, we have demonstrated that C_12_O_3_TR could enhance the membrane permeabilization of *P. digitatum* by using fluorescence microscopy in our previous study (Li et al., 2019). These results were similar with most studies which researched the mechanism of AMPs. For example, peptide MAF-1A could inhibit the growth of *Candida albicans* by changing the normal expression of genes which encoded ergosterol metabolism and fatty acid biosynthesis. And these pathways were related to the metabolic processes of cell membrane (Wang et al., 2017).

The cell wall is mainly composed by mannose glycoprotein, β-glucan and chitin. The RNA-Seq results also indicated that the cell wall of *P. digitatum* was affected by C_12_O_3_TR, due to the changes in fructose and mannose metabolism, amino sugar and nucleotide sugar metabolism (OuYang et al., 2016; Wang et al., 2018c). This finding was further confirmed by the changes of cell wall related enzymes activities, including β-1, 3-glucan synthase, chitinase and AKP (Figure 6). Similarly, many AMPs were found to be powerful to inhibit fungi growth by destroying the cell wall structure (Le et al., 2016; Wang et al., 2017). Cell wall is important in sustaining cell morphology and protecting cell against life threatening environmental conditions (Bowman and Free, 2006; Ruiz-Herrera et al., 2006). Chitin is one of the major macromolecule in the cell wall of filamentous fungi, and is very useful for the fungal development and pathogenicity (Klis et al., 2009). The CFW staining observation proved the effect of C_12_O_3_TR on the cell wall (Figure 5). CFW was widely used to determine the integrity of cell wall because of its preferential bounds to the chitin containing regions (Lewtak et al., 2014). In this study, the fewer and weaker blue fluorescence septa were observed under C_12_O_3_TR stress (Figure 5), indicating that the chitin content in the cell wall was influenced by C_12_O_3_TR treatment. This result was similar with the effect of antifungal proteins on cell wall chitin (Gandía et al., 2019). It was reported that the changes of chitin content was related to the activity of related enzymes, such as β-1, 3-glucan synthase and chitinase (OuYang et al., 2019). The present results showed that C_12_O_3_TR stress decreased β-1, 3-glucan synthase activity of *P. digitatum* but increased the chitinase activity (Figures 6A,B). The decrease of β-1, 3-glucan synthase activity lead to the decreased in the synthesis of glucan, and glucan was an essential precursor of chitin, therefore the chitin content was also reduced. On the other hand, chitinase was effective to catalyze the hydrolysis of chitin, thus the increase of chitinase activity also resulted in a sharp decline in chitin content. Moreover, AKP was an enzyme which was produced in the cytoplasm and usually located in the periplasmic space. If the permeability of cell wall was impaired, AKP would be released from fungal cells to the intercellular spaces (Yang et al., 2016). The increase of AKP activity further confirmed the damaged of the cell wall integrity by C_12_O_3_TR (Figure 6C).

Meanwhile, the metabolic processes of genetic information and energy were also found to be affected by C_12_O_3_TR stress (Table 2). But the effect on genetic information and energy metabolic process were only examined at transcriptional level. Further investigations were required to determine the anti-*P. digitatum* key mechanisms.

## Conclusion

Overall, the results from this study at transcriptional level revealed that C_12_O_3_TR was effective to inhibit *P. digitatum* growth through complex influences on *P. digitatum* metabolisms. And this study also observed the impairment on cell wall formation at superficial level. Further studies are still required to investigate the key anti-fungal mechanisms of C_12_O_3_TR against *P. digitatum* especially the genetic information and energy related metabolic processes.

## Data Availability Statement

The original contributions presented in the study are publicly available. This data can be found here: http://bigd.big.ac.cn/gsa/s/oUJ31ulZ.

## Author Contributions

KZ conceived and supervised the project. XL, GF, and WW designed the experiments and performed most of the experiments. XL analyzed the data and wrote the manuscript. LD and LY gave advises and edited the manuscript. All authors read and approved the final manuscript.

## Conflict of Interest

The authors declare that the research was conducted in the absence of any commercial or financial relationships that could be construed as a potential conflict of interest.

## Footnotes

1 http://fungi.ensembl.org/Penicillium_digitatum_pd1_gca_000315645/Info/Index

2 https://www.ncbi.nlm.nih.gov/genome/13384

3 http://www.genome.jp/kegg/

## Supplementary Material

TABLE S1 The primer sequence and log_2_^(fold change)^ value of DEGs.

TABLE S2 Profile of the objective transcriptome sequence data.

TABLE S3 The significant enrichment terms in the groups of C2 VS C_12_O_3_TR2.

TABLE S4 The significant enrichment terms in the groups of C12 VS C_12_O_3_TR12.

## References

[B1] Abedinzadeh M. Gaeini M. Sardari S. (2015). Natural antimicrobial peptides against *Mycobacterium tuberculosis*. *J. Antimicrob. Chemother.* 70 1285–1289. 10.1093/jac/dku570 25681127

[B2] Barad S. Sela N. Kumar D. Kumar-Dubey A. Glam-Matana N. Sherman A. (2016). Fungal and host transcriptome analysis of pH-regulated genes during colonization of apple fruits by *Penicillium expansum*. *BMC Genomics* 17:330. 10.1186/s12864-016-2665-7 27146851 PMC4855365

[B3] Bowman S. M. Free S. J. (2006). The structure and synthesis of the fungal cell wall. *Bioessays* 28 799–808. 10.1002/bies.20441 16927300

[B4] Ciociola T. Giovati L. Conti S. Magliani W. Santinoli C. Polonelli L. (2016). Natural and synthetic peptides with antifungal activity. *Future Med. Chem.* 8 1413–1433. 10.4155/fmc-2016-0035 27502155

[B5] Droby S. Eick A. Macarisin D. Cohen L. Rafaela G. Stange R. (2008). Role of citrus volatiles in host recognition, germination and growth of *Penicillium digitatum* and *Penicillium italicum*. *Postharvest Biol. Technol.* 49 386–396. 10.1016/j.postharvbio.2008.01.016

[B6] Gandía M. Garrigues S. Bolós B. Manzanares P. Marcos J. F. (2019). The myosin motor domain-containing chitin synthases are involved in cell wall integrity and sensitivity to antifungal proteins in *Penicillium digitatum*. *Front. Microbiol.* 10:2400. 10.3389/fmicb.2019.02400 31681248 PMC6813208

[B7] Jenssen H. Hamill P. Hancock R. E. W. (2006). Peptide antimicrobial agents. *Clin. Microbiol. Rev.* 19 491–511. 10.1128/CMR.00056-05 16847082 PMC1539102

[B8] Johnson E. T. Evans K. O. Dowd P. F. (2015). Antifungal activity of a synthetic cationic peptide against the plant pathogens *Colletotrichum graminicola* and three *Fusarium* species. *Plant Pathol. J.* 31 316–321. 10.5423/PPJ.NT.04.2015.0061 26361481 PMC4564158

[B9] Keymanesh K. Soltani S. Sardari S. (2009). Application of antimicrobial peptides in agriculture and food industry. *World J. Microbiol. Biotechnol.* 25 933–944. 10.1007/s11274-009-9984-7

[B10] Klis F. M. De Groot P. Hellingwerf K. (2009). Molecular organization of the cell wall of *Candida albicans*. *Med. Mycol.* 39 1–8. 10.1080/mmy.39.1.1.8-0 11800263

[B11] Lai T. Wang Y. Fan Y. Zhou Y. Bao Y. Zhou T. (2017). The response of growth and patulin production of postharvest pathogen *Penicillium expansum* to exogenous potassium phosphite treatment. *Int. J. Food Microbiol.* 244 1–10. 10.1016/j.ijfoodmicro.2016.12.017 28042969

[B12] Laverty G. McCloskey A. P. Gorman S. P. Gilmore B. F. (2015). Anti-biofilm activity of ultrashort cinnamic acid peptide derivatives against medical device-related pathogens. *J. Pept. Sci.* 21 770–778. 10.1002/psc.2805 26310860

[B13] Laverty G. Mclaughlin M. Shaw C. Gorman S. P. Gilmore B. F. (2010). Antimicrobial activity of short, synthetic cationic lipopeptides. *Chem. Biol. Drug Des.* 75 563–569. 10.1111/j.1747-0285.2010.00973.x 20374251

[B14] Le C. F. Gudimella R. Razali R. Manikam R. Sekaran S. D. (2016). Transcriptome analysis of *Streptococcus pneumoniae* treated with the designed antimicrobial peptides. DM3. *Sci. Rep.* 6:26828. 10.1038/srep26828 27225022 PMC4881017

[B15] Lewtak K. Fiołka M. J. Szczuka E. Ptaszyńska A. A. Kotowicz N. Kołodziej P. (2014). Analysis of antifungal and anticancer effects of the extract from *Pelargonium zonale*. *Micron* 66 69–79. 10.1016/j.micron.2014.06.001 24972056

[B16] Li X. Wang W. Liu S. Ruan C. Yi L. Deng L. (2019). Effects of the peptide H-OOWW-NH2 and its derived lipopeptide C12-OOWW-NH2 on controlling of citrus postharvest green mold. *Postharvest Biol. Technol.* 158 110979 10.1016/j.postharvbio.2019.110979

[B17] Lin J. Zhao X. Zhi Q. Zhao M. He Z. (2013). Transcriptomic profiling of *Aspergillus flavus* in response to 5-azacytidine. *Fungal Genet. Biol.* 56 78–86. 10.1016/j.fgb.2013.04.007 23644151

[B18] Liu J. Wang S. Qin T. Li N. Niu Y. Li D. Y. (2015). Whole transcriptome analysis of *Penicillium digitatum* strains treatmented with prochloraz reveals their drug-resistant mechanisms. *BMC Genomics* 16:855. 10.1186/s12864-015-2043-x 26499483 PMC4619488

[B19] Liu S. Wang W. Deng L. Yao S. Zeng K. (2019). Control of sour rot in citrus fruit by three insect antimicrobial peptides. *Postharvest Biol. Technol.* 149 200–208. 10.1016/j.postharvbio.2018.11.025

[B20] Liu Y. Yao S. Deng L. Ming J. Zeng K. (2019). Different mechanisms of action of isolated epiphytic yeasts against *Penicillium digitatum* and *Penicillium italicum* on citrus fruit. *Postharvest Biol. Technol.* 152 100–110. 10.1016/j.postharvbio.2019.03.002

[B21] Livak K. J. Schmittgen T. D. (2001). Analysis of relative gene expression data using real-time quantitative PCR and the 2 −ΔΔCT method. *Methods* 25 402–408. 10.1006/meth.2001.1262 11846609

[B22] López-García B. Harries E. Carmona L. Campos-Soriano L. López J. J. Manzanares P. (2015). Concatemerization increases the inhibitory activity of short, cell-penetrating, cationic and tryptophan-rich antifungal peptides. *Appl. Microbiol. Biotechnol.* 99 8011–8021. 10.1007/s00253-015-6541-1 25846331

[B23] Lu L. Ji L. Qiao L. Zhang Y. Chen M. Wang C. (2018). Combined treatment with *Rhodosporidium paludigenum*, and ammonium molybdate for the management of green mold in satsuma mandarin (Citrus unshiu Marc.). *Postharvest Biol. Technol.* 140 93–99. 10.1016/j.postharvbio.2018.01.005

[B24] Maget-Dana R. Peypoux F. (1994). Iturins, a special class of pore-forming lipopeptides: biological and physicochemical properties. *Toxicology* 87 151–174. 10.1016/0300-483x(94)90159-7 8160184

[B25] Mao X. Cai T. Olyarchuk J. G. Wei L. (2005). Automated genome annotation and pathway identification using the KEGG Orthology (KO) as a controlled vocabulary. *Bioinformatics* 21 3787–3793. 10.1093/bioinformatics/bti430 15817693

[B26] Marcet-Houben M. Ballester A. R. de la Fuente B. Harries E. Marcos J. F. González-Candelas L. (2012). Genome sequence of the necrotrophic fungus *Penicillium digitatum*, the main postharvest pathogen of citrus. *BMC Genomics* 13:646. 10.1186/1471-2164-13-646 23171342 PMC3532085

[B27] Moreno-Velásquez S. D. Seidel C. Juvvadi P. R. Steinbach W. J. Read N. D. (2017). Caspofungin-Mediated growth inhibition and paradoxical growth in *Aspergillus fumigatus* involve fungicidal hyphal tip lysis coupled with regenerative intrahyphal growth and dynamic changes in β-1, 3-Glucan synthase localization. *Antimicrob. Agents Chemother.* 61:e00710-17. 10.1128/aac.00710-17 28760907 PMC5610538

[B28] Mulder K. C. Lima L. A. Miranda V. J. Dias S. C. Franco O. L. (2013). Current scenario of people-based drugs: the key roles cationic antitumor and antiviral peptides. *Front. Microbiol.* 4:321. 10.3389/fmicb.2013.00321 24198814 PMC3813893

[B29] Muñoz A. López-García B. Pérez-Payá E. Marcos J. F. (2007). Antimicrobial properties of derivatives of the cationic tryptophan-rich hexapeptide PAF26. *Biochem. Biophys. Res. Commun.* 354 172–177. 10.1016/j.bbrc.2006.12.173 17222805

[B30] Nookaew I. Papini M. Pornputtapong N. Scalcinati G. Fagerberg L. Uhlén M. (2012). A comprehensive comparison of RNA-Seq-based transcriptome analysis from reads to differential gene expression and cross-comparison with microarrays: a case study in *Saccharomyces cerevisiae*. *Nucleic Acids Res.* 40 10084–10097. 10.1093/nar/gks804 22965124 PMC3488244

[B31] Omardien S. Brul S. Zaat S. A. J. (2016). Antimicrobial activity of cationic antimicrobial peptides against gram-positives: current progress made in understanding the mode of action and the response of bacteria. *Front. Cell Dev. Biol.* 4:111. 10.3389/fcell.2016.00111 27790614 PMC5063857

[B32] Osusky M. Zhou G. Q. Osuska L. Hancock R. E. Kay W. W. Misra S. (2000). Transgenic plants expressing cationic peptide chimeras exhibit broad-spectrum resistance to phytopathogens. *Nat. Biotechnol.* 18 1162–1166. 10.1038/81145 11062434

[B33] OuYang Q. Duan X. Li L. Tao N. (2019). Cinnamaldehyde exerts its antifungal activity by disrupting the cell wall integrity of *Geotrichum citri-aurantii*. *Front. Microbiol.* 10:55. 10.3389/fmicb.2019.00055 30761105 PMC6364577

[B34] OuYang Q. Tao N. Jing G. (2016). Transcriptional profiling analysis of *Penicillium digitatum*, the causal agent of citrus green mold, unravels an inhibited ergosterol biosynthesis pathway in response to citral. *BMC Genomics* 17:599. 10.1186/s12864-016-2943-4 27514516 PMC4982135

[B35] Palou L. Ali A. Fallik E. Romanazzi G. (2016). GRAS, plant-and animal-derived compounds as alternatives to conventional fungicides for the control of postharvest diseases of fresh horticultural produce. *Postharvest Biol. Technol.* 122 41–52. 10.1016/j.postharvbio.2016.04.017

[B36] Pan L. Zhao X. Chen M. Fu Y. Xiang M. Chen J. (2020). Effect of exogenous methyl jasmonate treatment on disease resistance of postharvest kiwifruit. *Food Chem.* 305:125483. 10.1016/j.foodchem.2019.125483 31610420

[B37] Petruzzelli R. Clementi M. Marini S. Coletta M. Di Stasio E. Giardina B. (2003). Respiratory inhibition of isolated mammalian mitochondria by salivary antifungal peptide histatin-5. *Biochem. Bioph. Res. Commun.* 311 1034–1040. 10.1016/j.bbrc.2003.10.104 14623286

[B38] Puig M. Moragrega C. Ruz L. Calderón C. E. Cazorla F. M. Montesinos E. (2016). Interaction of antifungal peptide BP15 with *Stemphylium vesicarium*, the causal agent of brown spot of pear. *Fungal Biol.* 120 61–71. 10.1016/j.funbio.2015.10.007 26693685

[B39] Romanazzi G. Feliziani E. Baños S. B. Sivakumar D. (2015). Shelf life extension of fresh fruit and vegetables by chitosan treatment. *Crit. Rev. Food Sci. Nutr.* 57 579–601. 10.1080/10408398.2014.900474 26047630

[B40] Ruiz-Herrera J. Elorza M. V. Valentín E. Sentandreu R. (2006). Molecular organization of the cell wall of *Candida albicans* and its relation to pathogenicity. *FEMS Yeast Res.* 6 14–29. 10.1111/j.1567-1364.2005.00017.x 16423067

[B41] Scocchi M. Mardirossian M. Runti G. Benincasa M. (2016). Non-membrane permeabilizing modes of action of antimicrobial peptides on bacteria. *Curr. Top. Med. Chem.* 16 76–88. 10.2174/1568026615666150703121009 26139115

[B42] Shah P. Hsiao F. S. Ho Y. H. Chen C. S. (2016). The proteome targets of intracellular targeting antimicrobial peptides. *Proteomics* 16 1225–1237. 10.1002/pmic.201500380 26648572

[B43] Shao X. Cheng S. Wang H. Yu D. Mungai C. (2013). The possible mechanism of antifungal action of tea tree oil on *Botrytis cinerea*. *J. Appl. Microbiol.* 114 1642–1649. 10.1111/jam.12193 23495848

[B44] Tao N. OuYang Q. Jia L. (2014). Citral inhibits mycelial growth of *Penicillium italicum* by a membrane damage mechanism. *Food Control* 41 116–121. 10.1016/j.foodcont.2014.01.010

[B45] Thery T. O’Callaghan Y. O’Brien N. Arendt E. K. (2018). Optimisation of the antifungal potency of the amidated peptide H-Orn-Orn-Trp-Trp-NH2 against food contaminants. *Int. J. Food Microbiol.* 265 40–48. 10.1016/j.ijfoodmicro.2017.10.024 29127809

[B46] Trapnell C. Williams B. A. Pertea G. Mortazavi A. Kwan G. van Baren M. J. (2010). Transcript assembly and quantification by RNA-Seq reveals unannotated transcripts and isoform switching during cell differentiation. *Nat. Biotechnol.* 28 511–515. 10.1038/nbt.1621 20436464 PMC3146043

[B47] Wang H. Lei Y. Yan L. Wan L. Ren X. Chen S. (2016). Functional genomic analysis of *Aspergillus flavus* interacting with resistant and susceptible peanut. *Toxins* 8:46. 10.3390/toxins8020046 26891328 PMC4773799

[B48] Wang L. Feng Z. Wang X. Wang X. Zhang X. (2010). DEGseq: an R package for identifying differentially expressed genes from RNA-seq data. *Bioinformatics* 26 136–138. 10.1093/bioinformatics/btp612 19855105

[B49] Wang T. Xiu J. Zhang Y. Wu J. Ma X. Wang Y. (2017). Transcriptional responses of *Candida albicans* to antimicrobial peptide MAF-1A. *Front. Microbiol.* 8:894. 10.3389/fmicb.2017.00894 28567034 PMC5434131

[B50] Wang W. Deng L. Yao S. Zeng K. (2018a). Control of green and blue mold and sour rot in citrus by the cationic antimicrobial peptide PAF56. *Postharvest Biol. Technol.* 136 132–138. 10.1016/j.postharvbio.2017.10.015

[B51] Wang W. Liu S. Deng L. Ming J. Yao S. Zeng K. (2018b). Control of citrus post-harvest green molds, blue molds, and sour rot by the Cecropin A-Melittin hybrid peptide BP21. *Front. Microbiol.* 9:2455. 10.3389/fmicb.2018.02455 30364142 PMC6191494

[B52] Wang Y. Feng K. Yang H. Zhang Z. Yuan Y. Yue T. (2018c). Effect of cinnamaldehyde and citral combination on transcriptional profile, growth, oxidative damage and patulin biosynthesis of *Penicillium expansum*. *Front. Microbiol.* 9:597. 10.3389/fmicb.2018.00597 29651282 PMC5884930

[B53] Yang S. Liu L. Li D. Xia H. Su X. Peng L. (2016). Use of active extracts of poplar buds against *Penicillium italicum* and possible modes of action. *Food Chem.* 196 610–618. 10.1016/j.foodchem.2015.09.101 26593534

[B54] Young M. D. Wakefield M. J. Smyth G. K. Oshlack A. (2010). Gene ontology analysis for RNA-seq: accounting for selection bias. *Genome Biol.* 11:R14. 10.1186/gb-2010-11-2-r14 20132535 PMC2872874

[B55] Zhou T. Wang X. Ye B. Shi L. Bai X. Lai T. (2018). Effects of essential oil decanal on growth and transcriptome of the postharvest fungal pathogen *Penicillium expansum*. *Postharvest Biol. Technol.* 145 203–212. 10.1016/j.postharvbio.2018.07.015

[B56] Zhou Y. Ma J. Xie J. Deng L. Yao S. Zeng K. (2018). Transcriptomic and biochemical analysis of highlighted induction of phenylpropanoid pathway metabolism of citrus in response to salicylic acid, *Pichia membranaefaciens*, and oligochitosan. *Postharvest Biol. Technol.* 142 81–92. 10.1016/j.postharvbio.2018.01.02

